# Sharp needle reconstructs peripheral outflow for patients with malfunctional arteriovenous fistula

**DOI:** 10.1080/0886022X.2024.2353351

**Published:** 2024-05-17

**Authors:** Yong Xu, Shu-Yuan Zhu, Yuan-Ming Li, Xin-Xin Liu, Hao Zhang, Lu-Fang Wang

**Affiliations:** a Department of Nephrology, The Third Xiangya Hospital, Hunan, China; b Department of Nephrology, Ningxiang People’s Hospital, Hunan, China; c Department of Nephrology, Changsha Jie-ao Hospital, Hunan, China

**Keywords:** Sharp needle, fistula malfunction, peripheral outflow reconstruction, extravascular shunt

## Abstract

**Objective:**

To investigate the feasibility and efficacy of combining ultrasound-guided sharp needle technique with percutaneous transluminal angioplasty (PTA) for treating outflow stenosis or dysfunction in arteriovenous fistula (AVF) among hemodialysis patients.

**Methods:**

From October 2021 to March 2023, patients with occluded or malfunctional fistula veins not amenable to regularly angioplasty were retrospectively enrolled in the study. They underwent ultrasound-guided sharp needle intervention followed by PTA. Data on the location and length between the two veins, technical success, clinical outcomes, and complications were collected. Patency rates post-angioplasty were calculated through Kaplan-Meier analysis.

**Results:**

A total of 23 patients were included. The mean length of the reconstructed extraluminal segment was 3.18 cm. The sharp needle opening was performed on the basilic vein (60.9%), brachial vein (26.1%), or upper arm cephalic vein (13%) to create outflow channels. Postoperatively, all cases presented with mild subcutaneous hematomas around the tunneling site and minor diffuse bleeding. The immediate patency rate for the internal fistulas was 100%, with 3-month, 6-month, and 12-month patency rates at 91.3%, 78.3%, and 43.5%, respectively.

**Conclusion:**

Sharp needle technology merged with PTA presents an effective and secure minimally invasive method for reconstructing the outflow tract, offering a new solution for recanalizing high-pressure or occluded fistulas.

## Introduction

As a ‘lifeline’ for hemodialysis patients, arteriovenous fistula (AVF) remains the most popular permanent access for hemodialysis, due to its low infection rate, few thrombosis events, low cost, and high long-term patency rate [1–3]. Stenosis and occlusion are common complications of AVF, which will affect the efficiency of dialysis and the prognosis of patients [4].

Currently, percutaneous transluminal angioplasty (PTA) is the primary management of malfunctional AVF, such as immature fistula [5], stenosis of fistula caused by neointimal hyperplasia [6,7], stenosis or occlusion of juxta-anastomosis [8], and AVF thrombosis [9].

Although the majority of AVF lesions can be treated by angioplasty, there remaining some cases, where wire failed to pass through the vein because of puncture damage or autogenous vein deficiency, etc. Surgical reconstruction such as proximal reconstruction, or graft implantation can salvage this situation [10–12]. However, surgery always consume vascular resources, leading to shortened vascular segments. Hence, we reported a novel strategy of AVF salvage by which a sharp needle was used to create a subcutaneous bypass between two fluent veins to maintain the AVF flow.

## Materials and methods

### Patient population

A total of 23 patients, whose fistula veins were occluded or lost – with or without thrombosis present – and in whom the guidewire could not pass during conventional PTA surgery, were retrospectively included in this study from October 2021 to March 2023. Consequently, they underwent recanalization with sharp needle therapy. Each patient met the following criteria: (1) The AVF was created between the cephalic vein of the forearm and the radial artery in patients undergoing regular intermittent hemodialysis; (2) Ultrasound or vascular imaging indicated an occluded pathway or the presence of only a perforating vein serving as the sole outflow tract, while central veins were free from significant stenosis or obstruction; (3) During hemodialysis, the blood flow rate was consistently below 180 mL/min, venous pressure was above 180 mmHg, or the AVF failed to meet the requirements for dialysis. The demographic characteristics of the patients are detailed in Table 1.

**Table 1.** The demographics of the 23 patients.

Characteristics	Patients(*n* = 23)
Sex (Men/Women)	11/12
Average age (years)	58.91(range 39–77)
Type of AVF	
Radial-Cephalic Fistula	23
Distance of two veins (cm)	3.18 (range 2–5)
Cause of end stage of renal disease	
Glomerulonephritis	7
Diabetes mellitus	10
Hypertension	4
Others*	2
Original outflow tract (%)	
Perforating vein (single outflow pathway)	16(69.6%)
Cephalic vein (stenosis or occluded)	6(26.1%)
Collateral circulation vessels	1(4.3%)

* Other including: nephrotic syndrome (1), and uncertain (1).

This study received approval from the Third Xiangya Hospital of Central South University. All procedures adhered to the ethical standards of the 1964 Declaration of Helsinki, as amended. Written informed consent was attained from every patient for procedures performed.

### Procedure of sharp needle recanalization and angioplasty

The procedures were performed under local anesthesia and an axillary block. The anesthetic mixture consisted of equal parts lidocaine (2%), ropivacaine (1%), and saline (0.9%).

### Preparation

**Figure 1.**
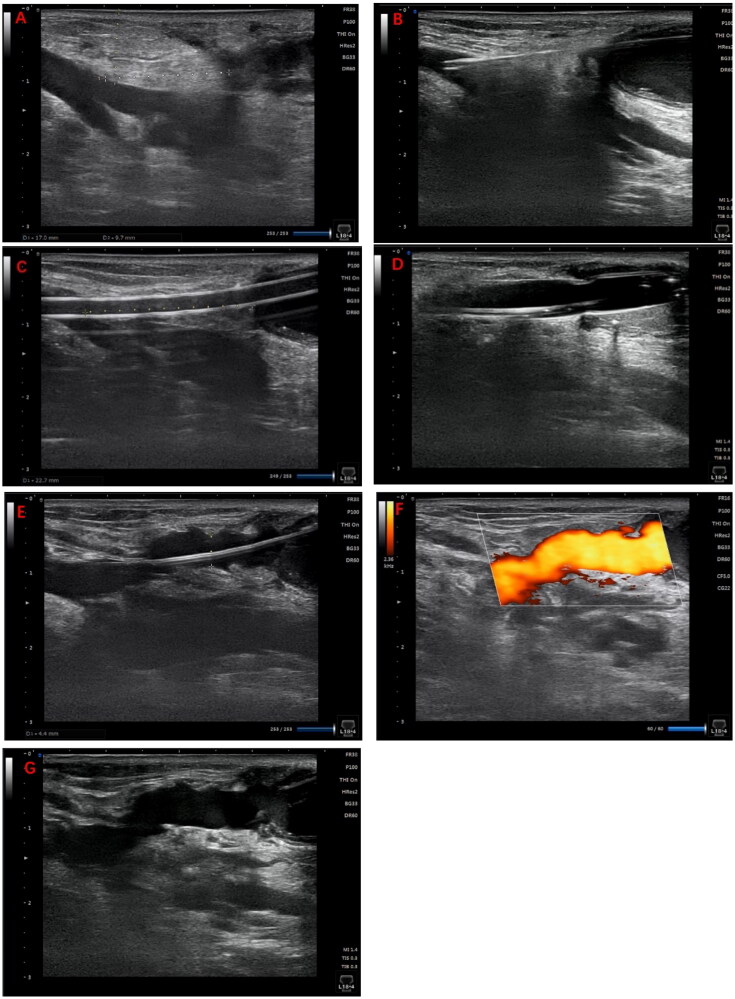
Illustrates the surgical procedure for sharp recanalization. (A) Using ultrasonography, the length and depth of the targeted vein are measured to devise the treatment plan. (B) An 18 G sharp needle is inserted through the fistula vein, penetrates the vessel wall, and advances through the subcutaneous tissue to reach the targeted vein. (C) A 6 F vascular sheath is introduced in stages. (D) A 6 mm × 4 cm balloon is inflated; (E–F) Ultrasonography is performed to ensure the vein is patent. (G) A subcutaneous hematoma is detected in the vicinity of the tunnel.

Ultrasonography was performed to locate patent veins and measure the distance and depth between the two vessels, facilitating the design of sharp needle recanalization, including the puncture point, direction and angle (Figure 1(A)).

### Sharp needle recanalization

In the case of non-thrombotic occlusion, the distal end of the occluded venous segment was selected as the puncture site. Under ultrasound guidance, an 18 G (64 mm length) puncture needle (TERUMO, Tokyo, Japan) was employed to perforate the vein. The needle was introduced into the subcutaneous tissue following the pre-planned direction and angle (Figure 1(B)). Subsequently, a 0.035-inch guidewire (TERUMO, Tokyo, Japan) and a 6 F vascular sheath were inserted, with the sheath advanced over the guidewire to access the intended vessel (Figure 1(C)). Ultrasound reassessment ensured that the percutaneous tunnel was supported by the sheath.

### Angioplasty

A 6 mm × 4 cm balloon (DK MEDTECH, Suzhou, China) was advanced over the guidewire to the subcutaneous segment of the vein. The balloon was incrementally inflated to its bursting pressure (average 26 ATM) and held at that level for 90 s (Figure 1(D)). Concurrently, appropriate pressure was exerted on the skin surface above the balloon to augment local subcutaneous pressure. Post-inflation, finger pressure was applied over the shunt to halt blood flow while the pressure within the balloon was slowly released, fostering the formation of a channel. This process was repeated three times to counteract potential channel recoil. Ultimately, ultrasonography was conducted once more to verify vascular patency and to screen for any complications (Figure 1(E–G)). Technical success was characterized by the unobstructed flow of blood into the outflow vein *via* the newly-created tunnel, with no leakage into the neighboring tissues.

### Hybrid operation

In cases with thrombosis, the approach to restoring blood flow was tailored to the size of the thrombus; this included urokinase thrombolysis or thrombectomy. Following the reestablishment of flow, a sharp technique was employed to open the outflow pathway, aided by balloon-assisted subcutaneous tunneling.

To minimize the risk of hemorrhagic complications, careful control of urokinase dosages – ranging from 100,000 to 200,000 units – was adopted. Once the outflow tract patency was reinstated, urokinase was systemically distributed rather than being confined to the previously occluded segment. This strategy effectively prevented the localized accumulation of urokinase during the reconstruction of the outflow tract. Patients with known contraindications to urokinase were excluded from this treatment approach.

As the final step in the procedure, a 6 F sheath was implanted at the distal end of the radial artery to address any stenosis or mural thrombus within the fistula vein.

### Statistical analysis

All statistical analyses were conducted using SPSS version 23.0. The paired sample t-test was applied to compare preoperative and postoperative values. To estimate the patency rates of the salvaged arteriovenous fistula (AVF), Kaplan-Meier analysis was utilized. A *p*-value below 0.05 was considered to indicate statistical significance.

## Results

### Clinical outcome

**Figure 2.**
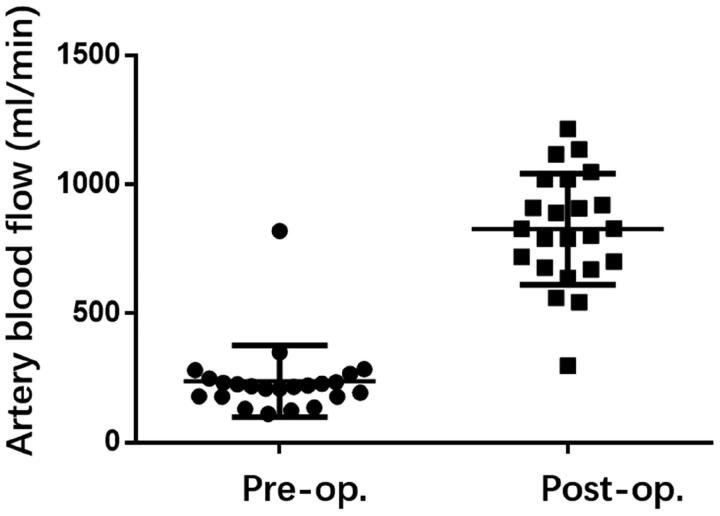
The brachial artery blood flow before and after the surgery.

**Figure 3.**
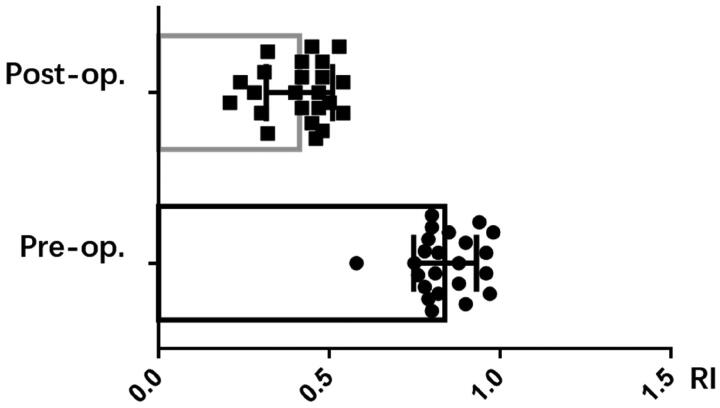
The RI before and after the surgery.

The clinical outcomes for 23 patients have been compiled in Table 2. The occluded or forfeited fistula veins were primarily located in the region stretching from the forearm to the elbow, and 8 instances featured distal thromboses. Three patients were subjected to hybrid surgical interventions – with one undergoing thrombectomy and two treated with urokinase. Balloons measuring either 6 mm × 4 cm or 6 mm × 6 cm were utilized to widen the subcutaneous tunnel. Following dilation, the mean diameter of the subcutaneous tunnel was 2.94 mm, and the average length was 3.18 cm, as reported in Table 2. The fistula’s immediate postoperative patency rate achieved 100%, coupled with an average brachial artery flow rate of 827.8 mL/min and a resistive index (RI) of 0.41, as shown in Figures 2 and 3.

**Table 2.** Reconstruction outflow between two veins.

Parameter	Outcome
Location of occlusion (*n*, %)	
mid-forearm cephalic vein	5 (21.7%)
perforating vein	16 (69.6%)
accessory cephalic vein	2 (8.7%)
Connecting vessels as outflow (*n*, %)	
Basilic	14 (60.9%)
Brachial vein (deep vein)	6 (26.1%)
Upper arm cephalic	3 (13.0%)
Distance of two veins (cm)	3.18 (range 2–5)
Balloon sizes	6mm × 4 cm or 6 mm × 6 cm
Subcutaneous tunnel diameter*	2.94 ± 0.57 mm (2.0–4.5 mm)
Resistance Index, RI	Pre-operation: 0.839 ± 0.092
	Post-operation: 0.413 + 0.097
	*p* < 0.001
Brachial artery blood flow	Pre-operation:237.913 ± 138.614 mL/min
	Post-operation:827.826 ± 215.879 mL/min
	*p* < 00.001

* Size at the smallest point of the tunnel segment diameter.

### Risks and complications

After treatment, mild subcutaneous hematoma around the tunnel and slightly diffuse bleeding were detected in all cases, with the largest size of 32 mm × 40 mm × 8 mm. We did not measure the remaining small hematomas and no active bleeding was detected. All hematoma was absorbed in 1–2 weeks. Two patients had pseudoaneurysm above the tunnel, which was effectively compressed by a balloon. One case experienced local subcutaneous infection, which was effectively treated with local (Mupirocin ointment) and systemic antibiotics therapy (500 mg Amoxicillin orally twice daily for 7 days). Although valve-like structures were found in the lumens of fistula in four patients (17.4%), which might impede blood flow, they did not affect immediate patency.

### Follow-up

**Figure 4.**
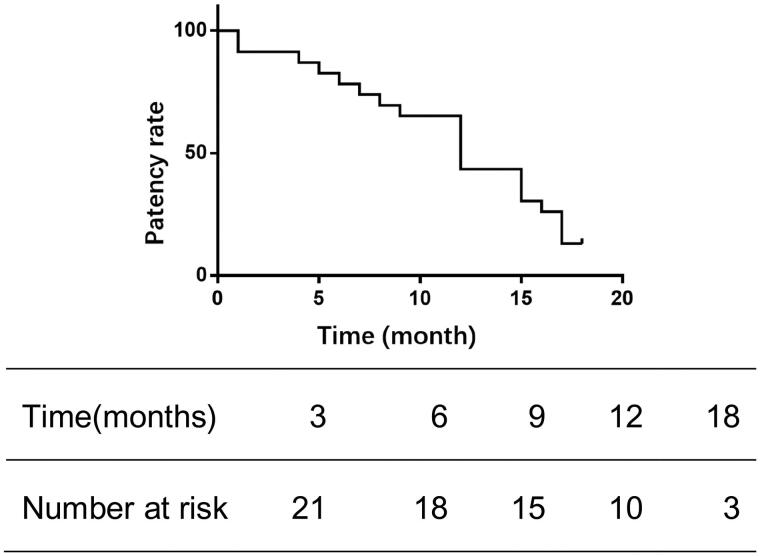
Kaplan-Meier curve of fistulas patency.

**Figure 5.**
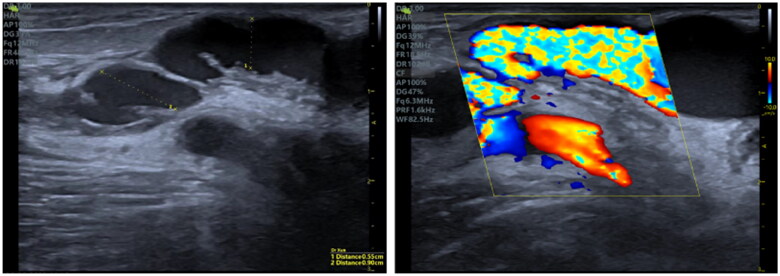
Two weeks after treatment, a paravalvular stenosis in subcutaneous tunnel was detected in a 70-years-old male patient, which eventually progressed to thrombosis 5 months later.

The follow-up process was carried out *via* telephone and during outpatient visits. The median duration of follow-up was 11 months, with a range from 1 to 18 months. Telephone follow-up deemed cases as effective when the patient received 2–3 dialysis sessions weekly, with a dialysis blood flow rate exceeding 220 mL/min, and venous pressure below 180 mmHg. Outpatient follow-up considered cases effective if the brachial artery blood flow was above 500 mL/min and there was an absence of significant stenosis. The patency rates at 3, 6, and 12 months were 91.3%, 78.3%, and 43.5%, respectively, as shown in Figure 4. Additionally, paravalvular stenosis within the tunnel, as seen in Figure 5, was observed in three patients. Thrombi formed in the subcutaneous channel in three patients, occurring at 5, 7, and 6 months, respectively.

## Discussion

Stenosis or occlusion of the outflow pathway can compromise fistula function, impeding adequate hemodialysis for patients. Central vein occlusions may occur due to indwelling catheters [13], while peripheral vein occlusions can be caused by stenosis, thrombosis, and congenital vascular malformation [14]. These complications can result in a poor prognosis for patients requiring regular hemodialysis.

Sharpened needle therapy has seen widespread use in managing central vein occlusions. Arabi et al. [15] reported six cases where a radiographically guided sharp needle was used to penetrate the occlusion, recanalizing the superior vena cava and either AVF patency or serving as a base for dialysis catheter placement. Similarly, Yang et al. used a stiff guidewire to perforate the occlusion from the femoral vein through a jugular vein [16]. Furthermore, Murphy et al. [17] and Gupta et al. [18] detailed the creation of an extravascular shunt in the central veins *via* needle puncture, maintaining patency in the right subclavian and brachiocephalic veins for three months and offering insight into non-luminal pathways for treating venous chronic occlusions related to dialysis.

Peripheral blood vessels, being more superficial and having multiple collateral pathways, offer opportunities to reconnect occluded outflow pathways. In this study, we combined sharp needle technique with PTA to link adjacent patent veins and reconstruct the outflow pathway. The primary patency rate was 100% (23/23), with a significant improved brachial artery blood flow, which increased from 237 mL/min to 827 mL/min after treatment (*p* < 0.001), and a significant reduced RI.

The inter-vessel connectivity distance is limited by the puncture needle’s length, hence, ultrasound plays a pivotal role in selecting a target vessel within a suitable range and in the strategic design of the tunnel trajectory. It’s worth noting that peripheral outflow occlusions frequently occur in the forearm, elbow region, or at a single venipuncture site. Our study highlights that the ideal candidate veins for AVF reconstruction were the basilic (60.9%), the brachial (26.1%), and the upper arm cephalic vein (13.0%), which was consistent with previous reports by Miller [19]; their proximity to problematic veins and ease of access provide a tangible advantage. The target vein’s diameter also contributes to long-term patency [20], with a diameter greater than 3.5 mm favoring recanalization success. Furthermore, excessive shear stress, caused by high blood flow, can impact fistula lifespan [21], making the angle of the new tunnel-vessel junction critical for patency, although the optimal angle remains undetermined.

Balloon dilation is integral to forming a new tunnel. Our technique involved using a balloon that matched the diameter of the target vessel and inserting it into the tunnel, applying surface pressure to minimize hematoma development. We maintained the inflation for 90 s, ensuring successful vessel dilation. Our method mirrors that of Noh et al. [22], who also reported effective dilation with minimal complications.

The average tunnel diameter measured 2.94 mm immediately following the procedure, and a 43.5% patency rate was observed at the 12-month follow-up. In three patients, a ‘valve-like structure’ developed at the tunnel’s initiation point (Figure 5), triggering thrombosis – likely an effect of repeated punctures or a single outflow site. This suggests that intimal hyperplasia may be responsible [13,23,24], warranting further investigation into intraluminal support as a potential solution.

Despite thrombolysis with urokinase yielded positive results in two patients, without significant hematomas or bleeding, the limited number of subjects is a notable limitation of the study. It has been established that stent graft implantation post-surgery enhances success rates and decreases complications. However, due to economic constraints and the tunnel typically passing through the transverse cubital crease, stent implantation post-recanalization was not performed in this study. Additionally, the tunnel segment was not used for the puncture approach.

To sum up, sharp opening for vessel connection followed by tunnel formation and the maintenance of patency through balloon dilatation is a safe, and cost-effective technique, which provided a novel strategy for the treatment of malfunctional AVF.
